# A Sliding Microfluidic Chip-Integrated Colorimetric Biosensor Using MnO_2_ Nanoflowers for Rapid *Salmonella* Detection

**DOI:** 10.3390/mi16080904

**Published:** 2025-07-31

**Authors:** Yidan Niu, Juntao Jiang, Xin Zhi, Jiahui An, Yuhe Wang

**Affiliations:** 1 College of Information and Electrical Engineering, China Agricultural University, Beijing 100083, China; 2022307150216@cau.edu.cn; 2 College of Engineering, China Agricultural University, Beijing 100083, China; 1371201957@cau.edu.cn; 3 College of Science, China Agricultural University, Beijing 100083, China; 2022307150401@cau.edu.cn (X.Z.); 2022307150202@cau.edu.cn (J.A.)

**Keywords:** colorimetric biosensor, microfluidic chip, manganese dioxide nanoflowers, *Salmonella* detection

## Abstract

Rapid screening of foodborne pathogens is critical for food safety, yet current detection techniques often suffer from low efficiency and complexity. In this study, we developed a sliding microfluidic colorimetric biosensor for the fast, sensitive, and multiplex detection of *Salmonella*. First, the target bacteria were specifically captured by antibody-functionalized magnetic nanoparticles in the microfluidic chip, forming magnetic bead–bacteria complexes. Then, through motor-assisted sliding of the chip, manganese dioxide (MnO_2_) nanoflowers conjugated with secondary antibodies were introduced to bind the captured bacteria, generating a dual-antibody sandwich structure. Finally, a second sliding step brought the complexes into contact with a chromogenic substrate, where the MnO_2_ nanoflowers catalyzed a colorimetric reaction, and the resulting signal was used to quantify the *Salmonella* concentration. Under optimized conditions, the biosensor achieved a detection limit of 10 CFU/mL within 20 min. In spiked pork samples, the average recovery rate of *Salmonella* ranged from 94.9% to 125.4%, with a coefficient of variation between 4.0% and 6.8%. By integrating mixing, separation, washing, catalysis, and detection into a single chip, this microfluidic biosensor offers a user-friendly, time-efficient, and highly sensitive platform, showing great potential for the on-site detection of foodborne pathogens.

## 1. Introduction

Rapid detection of foodborne pathogens is crucial for ensuring food safety. According to data from the World Health Organization (WHO), approximately 600 million people worldwide suffer from foodborne illnesses each year, resulting in an estimated 420,000 deaths [1]. Early detection and warning are essential to prevent the widespread transmission of these pathogens, underscoring the urgent need for detection methods that are rapid, accurate, and user-friendly.

Conventional detection techniques such as plate counting, polymerase chain reaction (PCR), and enzyme-linked immunosorbent assay (ELISA) are widely used but have inherent limitations [2,3]. Plate counting is considered the gold standard for viability assessment but is time-consuming, typically requiring 24–48 h to produce results [4]. PCR offers high sensitivity and specificity; however, it involves complex and labor-intensive nucleic acid extraction procedures [5]. While ELISA is a highly sensitive method in many contexts, its detection limit may still be insufficient for trace-level or early-stage pathogen detection compared to amplification-based techniques [6]. These limitations constrain the use of conventional methods in rapid and field-deployable applications [7].

To address these challenges, microfluidic chip technology has gained increasing attention in recent years due to its advantages of integration, miniaturization, and automation [8]. Microfluidic platforms can integrate sample pretreatment, bacterial enrichment, and signal detection within a single device, greatly improving analytical throughput, portability, and detection efficiency [9]. Among the various types of microfluidic systems, sliding microfluidic chips provide a power-free solution for liquid manipulation, such as droplet segmentation, mixing, and reaction. These operations are performed via simple sliding motions, making these systems highly suitable for use in resource-limited settings [10]. Guo reported a slide-type microfluidic chip for low-cost, power-free detection of Salmonella, though the overall system design is relatively complex [11]. Xue reported a SlipChip-based colorimetric biosensor for the power-free detection of *Salmonella*, featuring integrated fluid control and signal amplification via Au@PtPd nanozymes, with a compact design but involving multiple fluidic steps that may increase operational complexity [12]. These studies highlight the promise of sliding microfluidic platforms in enabling low-cost and power-free pathogen detection, while also revealing that the integration of multiple fluidic functions can increase system complexity and operational requirements.

Immunomagnetic separation (IMS) has also emerged as a powerful technique for selectively capturing and enriching target pathogens, thereby enhancing detection accuracy and sensitivity [13]. However, conventional IMS workflows typically require multiple manual steps and external equipment, which limit their practicality in decentralized or point-of-care settings [14]. Integrating IMS with microfluidic platforms has been shown to reduce operational complexity while maintaining effective pathogen isolation, offering a promising strategy for portable biosensing applications [15]. Rodríguez reported a microfluidic platform integrating continuous magnet-based separation and fluidic control for efficient purification of magnetic particles, offering a promising foundation for immunomagnetic applications compared to segmented-magnet systems [16]. Hu reported a scalable microfluidic method leveraging collective transport for high-throughput magnetic particle separation, paving the way for large-volume immunomagnetic applications such as blood purification and environmental remediation [17]. Collectively, these studies underscore the potential of microfluidic integration to overcome the limitations of conventional immunomagnetic separation, enabling more efficient, scalable, and field-adaptable biosensing platforms.

In terms of signal transduction, natural enzymes are commonly employed in biosensors due to their excellent catalytic efficiency and substrate specificity [18]. However, their high cost, poor storage stability, and susceptibility to environmental factors such as temperature and pH severely limit their practical use [19]. To overcome these issues, artificial nanozymes have been developed as robust and cost-effective alternatives [20]. Among them, manganese dioxide (MnO_2_) nanoflowers stand out for their three-dimensional hierarchical structure, large specific surface area, superior catalytic activity, and good biocompatibility [21]. Unlike natural enzymes, MnO_2_ nanoflowers maintain stable catalytic activity under harsh environmental conditions, including extreme pH and temperature, making them especially suitable for on-site detection in complex sample matrices [22]. Cui reported a dual-mode biosensor that employs MPDA/MnO_2_ nanozymes with tunable oxidase-like activity for specific detection of Staphylococcus aureus, demonstrating high sensitivity and leveraging nanozyme-based signal amplification [23].

**Figure 1:**
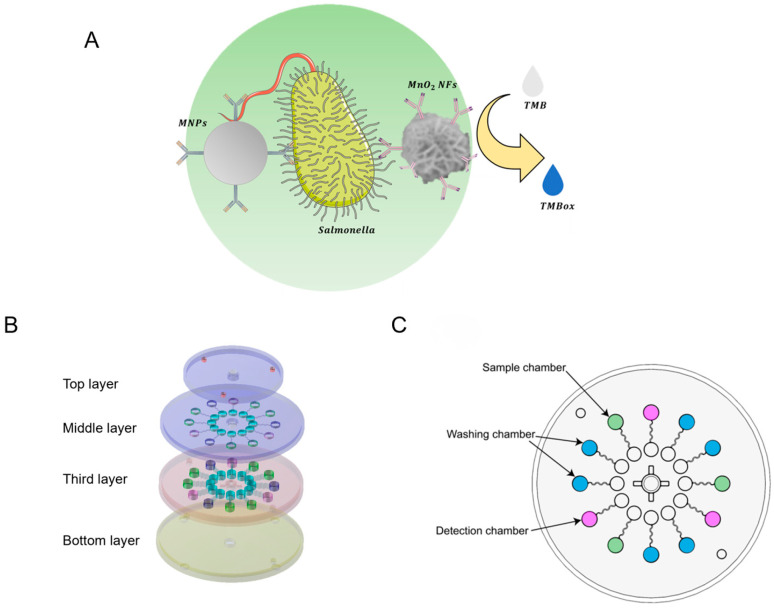
Principle of the biosensor: (**A**) biosensing principle; (**B**) structure of the sliding microfluidic chip; (**C**) radial arrangement of functional chambers in the microfluidic device.

Despite these advances, many existing biosensing systems still involve relatively complex fluidic operations or are limited in throughput and adaptability to resource-limited settings. In particular, platforms integrating both immunomagnetic separation and nanozyme-based signal amplification often require multiple manual handling steps, limiting their potential for rapid, multiplexed detection. Unlike conventional multiwell-based assays that involve repeated pipetting, magnetic transfers, and reliance on benchtop instruments, the proposed device enables transfer-free immunoassays with fixed magnetic positioning and smartphone-based readout, thereby reducing loss, enhancing reproducibility, and facilitating on-site application. To overcome these challenges, we developed a power-free sliding microfluidic biosensor that seamlessly integrates immunomagnetic separation with MnO_2_ nanozyme-mediated colorimetric detection (Figure 1). The device features a multichannel design and a semi-automated sliding mechanism, enabling the sequential execution of target capture, washing, catalytic reaction, and signal readout in a compact and user-friendly format. This platform achieves improved sensitivity, operational simplicity, and parallel sample processing, offering a cost-effective and field-deployable solution for the rapid screening of foodborne pathogens.

## 2. Materials and Methods

### 2.1. Bacterial Culture

*Salmonella typhimurium* (ATCC 14028) was used as the target pathogen, while *Listeria monocytogenes* (ATCC 13932), *Escherichia coli* 157:H7 (ATCC 43888), and *Staphylococcus aureus* (CICC 10001) served as non-target pathogens. All strains were stored in glycerol/agar medium (50%, *v*/*v*) at −20 °C for future use. Each bacterial culture was first inoculated into 5 mL of sterile Luria–Bertani (LB) medium and incubated with shaking at 37 °C for 16–18 h to obtain bacterial suspensions with concentrations of approximately 10^9^ CFU/mL, which were confirmed through standard plate counting. Serial 10-fold dilutions with sterile PBS were performed to prepare bacterial samples with concentrations ranging from 10^1^ to 10^6^ CFU/mL.

### 2.2. Antibody Conjugation on Immunomagnetic Beads

Carboxylated magnetic beads were modified with anti-*Salmonella* polyclonal antibodies using the EDC-NHSS method. First, a mixture of 100 µL EDC (10 mg/mL) and 100 µL NHSS (10 mg/mL) was added to 900 µL of PB (0.01 M, pH 6.0) containing carboxylated magnetic beads, followed by shaking for 1 h. After two washes with PB6.0 via magnetic separation, the beads were resuspended in 1 mL of PB (0.01 M, pH 7.4), and 50 µg of antibodies was added for 2 h of incubation. Finally, 50 µL of skim milk (20 mg/mL) was added to block the beads for 4 h. After magnetic separation and washing, the immunomagnetic beads were obtained.

### 2.3. Design and Fabrication of the Microfluidic Chip

The microfluidic chip was a key component of this biosensor. The chip consisted of four layers (see Figure 1 for details): The first layer (diameter: 64 mm; thickness: 4 mm) contained four chambers for accommodating magnets. The second layer (diameter: 82 mm; thickness: 3 mm) featured a central through-hole and a set of functional chambers and channels distributed across three quadrants. The third layer (diameter: 82 mm; thickness: 4 mm) included twelve air chambers and channels matching the functional chambers of the second layer. The bottom layer housed the components for fixing the stepper motor.

The chip mold was designed using SolidWorks 2022 software (Concord, MA, USA) and fabricated using an 3D printer (Object 24, Stratasys, Eden Prairie, MN, USA). The chip was then produced via PDMS casting.

**Figure 2:**
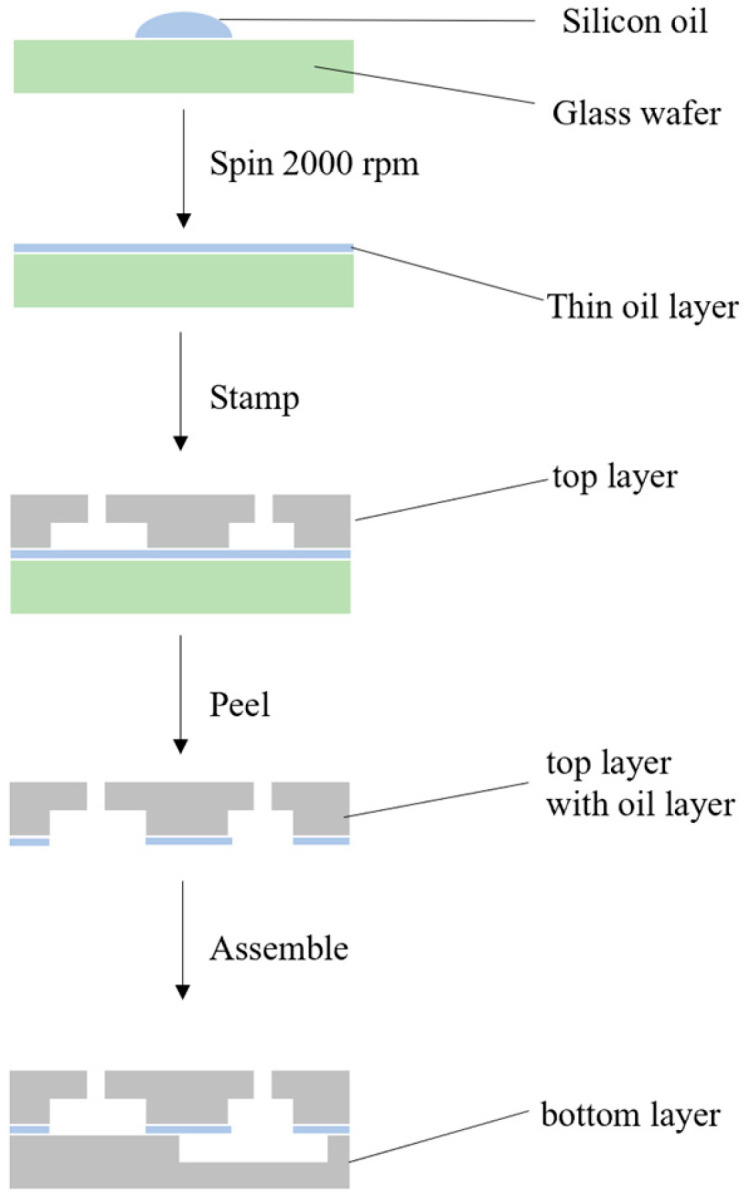
The assembly diagram of the chip.

As illustrated in Figure 2, the bottom layer was first secured to the stepper motor using four copper bolts. The third layer was then aligned and mounted onto the bottom layer via two cylindrical alignment holes, with a 3D-printed hollow connecting shaft inserted into the motor’s rotating axis. After positioning the third layer, the second layer—coated with silicone oil—was placed on top, and a solid cylindrical connector was inserted through the central cross-hole. Finally, the top layer was coaxially installed onto the second layer, ensuring that the magnet chambers were positioned directly above the washing chambers. All four layers were assembled concentrically along the motor’s rotating axis. Additionally, a small vent hole was drilled at the top of each buffer chamber to allow air expulsion when the pneumatic chambers were compressed.

### 2.4. Implementation of Automated Detection

An Arduino microcontroller was used to drive a two-phase, four-wire stepper motor. Custom control v1.0 (Testing Phase-Not for Production) software was developed using MIT App Inventor 2.0 to regulate the motor’s rotation. By rotating the chip via the stepper motor, the double-antibody sandwich complexes were transferred between chambers, enabling sequential mixing, washing, and colorimetric reactions.

Prior to implementing automated control, manual sliding tests were conducted to verify the feasibility of key fluidic operations such as mixing and chamber alignment. Based on the favorable preliminary results, a stepper motor was subsequently integrated to enable precise, consistent, and semi-automated control of the chip’s sliding motion.

### 2.5. Synthesis of MnO_2_-NH_2_ Nanoflowers

A solution of 1.58 g potassium permanganate (KMnO_4_) in 100 mL deionized water (0.1 M) was mixed with 0.0261 g polyvinylpyrrolidone (PVP, dissolved in 10 mL deionized water) under stirring. The mixture was heated to 90 °C in a water bath, followed by the addition of 100 mL of hydrochloric acid (0.2 M). After 1 h of continued heating, a dark brown precipitate formed. The product was cooled to room temperature, washed three times with ultrapure water (10,000 rpm, 10 min per centrifugation), and resuspended in ultrapure water for storage at 4 °C.

Next, 10 mg of MnO_2_ nanoflowers were centrifuged (10,000 rpm, 5 min) to remove excess water and washed three times with anhydrous ethanol. The pellet was resuspended in 2 mL ethanol, mixed with 1 mL (3-aminopropyl) triethoxysilane (APTES, 99% wt%), and incubated at 37 °C for 12 h with shaking. After sequential washing with ethanol and deionized water (twice each, 10,000 rpm, 5 min), the product was resuspended in 10 mL ultrapure water to a final concentration of 1 mg/mL, yielding amino-functionalized MnO_2_ nanoflowers (MnO_2_-NH_2_), consistent with previous reports using the same synthesis method [24].

### 2.6. Synthesis of Ab-MnO_2_ Probes

To prepare antibody-conjugated MnO_2_ nanoflowers, 2 mL glass vials were first rinsed three times with 1 mL of phosphate buffer (PB6.0, 0.01 M, pH 6.0). Subsequently, the following reagents were sequentially added: 0.5 mL of amino-functionalized MnO_2_ nanoflowers (1 mg/mL in PB6.0), 25 μg of non-biotinylated monoclonal antibody (1 mg/mL in PB6.0), 50 μL of EDC (1 mg/mL in PB6.0), and 50 μL of NHSS (1 mg/mL in PB6.0). The mixture was incubated at room temperature for 2 h to allow for EDC/NHSS-mediated covalent conjugation.

After activation and conjugation, 75 μL of skim milk and an additional 50 μL of EDC (1 mg/mL in PB6.0) were added for blocking and stabilization. The reaction was allowed to proceed for 1 h. The resulting conjugates were then washed three times with ultrapure water (centrifugation at 10,000 rpm for 5 min each time) and finally resuspended in 0.5 mL of specialized storage buffer (1 mg/mL). The conjugated nanoflowers were stored at 4 °C until further use.

### 2.7. Detection Procedure

The complete detection process consisted of six main steps: sample loading, immunocapture, magnetic washing, labeling, colorimetric detection, and signal readout. First, 100 μL of bacterial suspension (prepared in PBS), 14 μL of antibody-functionalized magnetic beads (1 mg/mL), and 14 μL of MnO_2_ nanozyme solution (1 mg/mL) were sequentially introduced into the sample chamber of the sliding chip using a micropipette. The chip was then operated using the stepper motor to perform repeated sliding cycles while applying magnetic actuation to vertically oscillate the magnetic nut for mixing and immunocomplex formation.

After incubation, the nut was magnetically immobilized and transferred sequentially into two washing chambers, each containing 100 μL of PBS. The same magnetic oscillation was applied for 30 s per chamber to remove unbound materials. The nut was then guided into the detection chamber containing 100 μL of TMB substrate solution. The MnO_2_ nanozymes catalyzed TMB oxidation to generate a blue color signal within 10 min. Finally, the chip was placed in a white background enclosure, and the color intensity was recorded using a smartphone camera. The images were analyzed via a custom app to obtain semi-quantitative results based on blue channel intensity.

For validation in real sample matrices, fresh pork loin was sourced from a certified retail supplier in Beijing, China. A 25 g portion of pork was homogenized with 225 mL of sterile phosphate-buffered saline (PBS) using a BagMixer 400 (Beijing HYCY Technology Development Co., Ltd., Xizhaosi Middle Street No. 4, Chongwen District, Xinghai Hongchang Building Room A305, Beijing, China) for 4 min. The homogenate was filtered through a 0.45 μm membrane and allowed to stand for 15 min to settle particulates. The resulting supernatant was then spiked with *Salmonella* at defined concentrations ranging from 6.4 × 10^1^ to 2.5 × 10^5^ CFU/mL and subjected to the above-described detection workflow.

## 3. Results

### 3.1. Working Principle of the Biosensor

**Figure 3:**
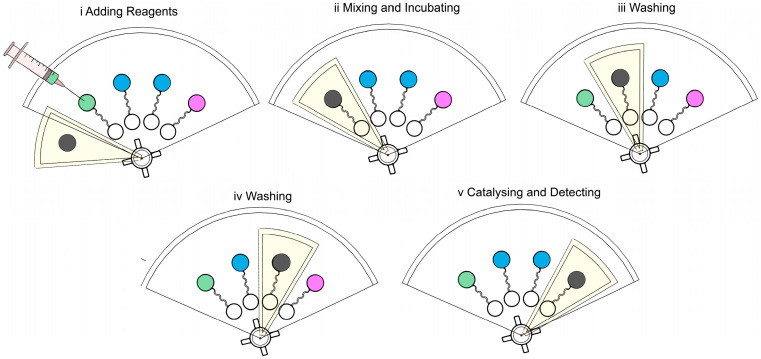
Working principle of the sliding microfluidic chip.

To achieve efficient and portable detection of foodborne pathogens, we designed a sliding microfluidic biosensor that integrates immunomagnetic separation with MnO_2_ nanozyme-based colorimetric signal amplification. As illustrated in Figure 3, the detection process consists of a sequence of magnetically controlled fluidic operations within a multilayer microchip. Initially, the bacterial sample, immunomagnetic beads, and MnO_2_ nanoflowers were co-introduced into the sample chamber via a syringe. A rotating magnetic layer was then used to position an external magnet above the chamber, attracting the embedded magnetic nut upward. When the magnet was removed, the nut fell back due to gravity. This controlled vertical oscillation facilitated efficient mixing and promoted the formation of sandwich immunocomplexes between the magnetic beads, target bacteria, and nanozymes.

Following the incubation step, the nut was immobilized using the magnet and gently immersed in the solution to capture the formed complexes on its surface. The chip was subsequently rotated to sequentially transfer the nut into two washing chambers, where residual unbound materials were removed through similar magnetic actuation. After washing, the complexes were moved into the final detection chamber containing TMB substrate. The MnO_2_ nanoflowers catalyzed the oxidation of TMB, resulting in a visible color change due to their intrinsic oxidase-like activity. The intensity of the colorimetric signal was recorded and analyzed using a smartphone App, enabling rapid and semi-quantitative detection. This compact, power-free platform allows integrated sample processing, target enrichment, and signal readout in a single device, offering a robust and field-deployable solution for on-site pathogen screening.

### 3.2. Simulations of the Magnetic Field

**Figure 4:**
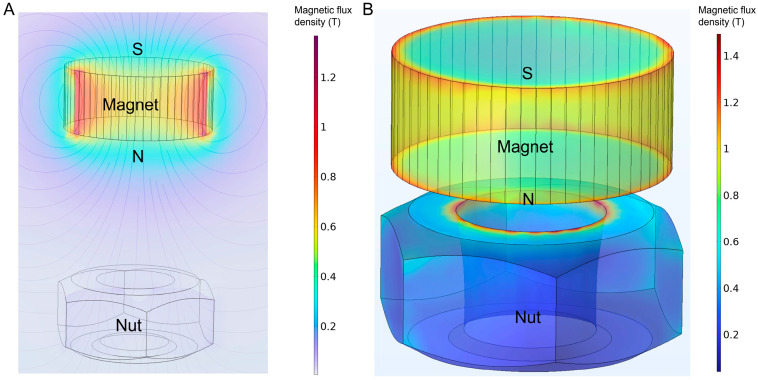
Simulations of the magnetic field. (**A**) Magnetic field distribution during magnet-nut separation; (**B**) Magnetic field distribution during magnet-nut adhesion.

The magnetic field distribution during nut actuation and magnetic separation was simulated using COMSOL 6.2 and is shown in Figure 4. In Figure 4A, the permanent magnet is placed above the nut to simulate the magnetic actuation stage. A strong vertical magnetic flux is observed, concentrated along the central axis of the magnet. The magnetic flux density in the region between the magnet and the nut reaches approximately 0.6 to 0.8 T, generating an upward magnetic force sufficient to lift the nut from the chamber bottom. The calculated magnetic force in this configuration is approximately 7 mN, which exceeds the gravitational force acting on the nut (approximately 4.6 mN). This confirms that the magnet can reliably attract and reposition the nut during its cyclic motion in the mixing process.

In Figure 4B, the magnetic flux distribution around the nut is further visualized under the same magnet-on-top configuration. The field lines converge into the upper surface and edges of the nut, forming a high-gradient region suitable for magnetic particle capture. The flux density near the nut surface exceeds 1.0 T, providing strong local magnetic forces for the retention of immunomagnetic bead complexes during the washing and detection steps. These simulation results validate the effectiveness of the magnetic design in enabling both mechanical actuation of the nut and stable magnetic separation of target-bound beads, supporting the dual-functionality of the system.

### 3.3. Optimization of Parameters

#### 3.3.1. Optimization of Immunomagnetic Bead Volume

**Figure 5:**
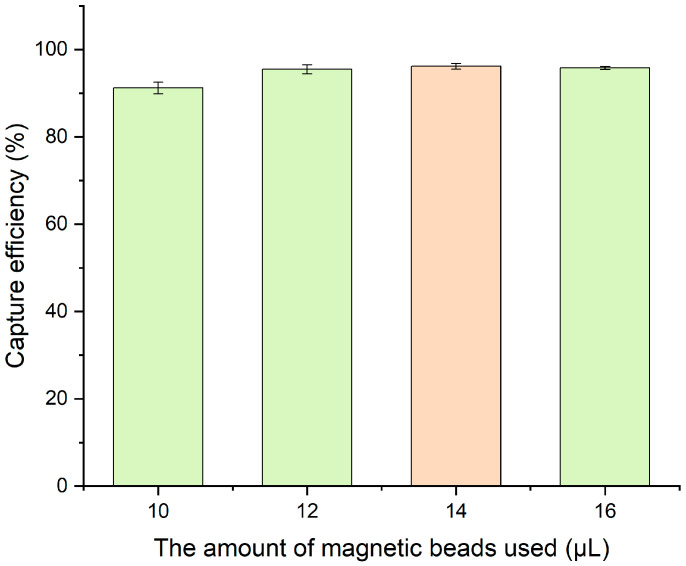
Optimization of magnetic bead usage. Error bars represent standard deviations from three independent measurements (N = 3).

The amount of magnetic beads plays a critical role in biosensor performance. In this study, 180 μm immunomagnetic beads synthesized at a concentration of 1 mg/mL were used to capture target bacteria at a concentration of 10^5^ CFU/mL. Various bead volumes were tested, and capture efficiency was determined based on the residual bacteria in the supernatant. As shown in Figure 5, increasing the bead volume from 8 μL to 14 μL improved the capture efficiency from 92.50% to 95.63%. However, further increases in volume resulted in no significant improvement. Therefore, 14 μL was selected as the optimal bead volume for subsequent experiments.

#### 3.3.2. Optimization of MnO_2_ NF Volume

**Figure 6:**
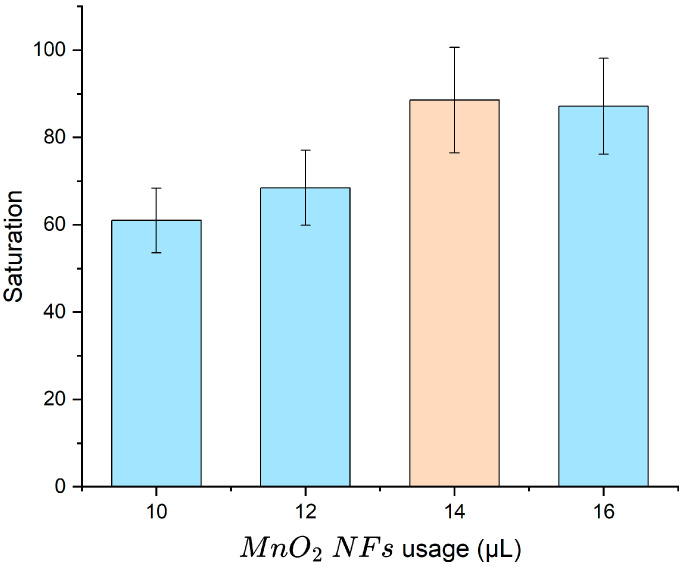
Optimization of MnO_2_ NF usage. Error bars represent standard deviations from three independent measurements (N = 3). Saturation (S) refers to the normalized absorbance of oxidized TMB, calculated as OD_450nm_/OD_max_, where OD_max_ = 1.0 (the plateau value at high bacterial concentration).

The observed signal is attributed to the intrinsic oxidase-like activity of MnO_2_ nanoflowers, which catalyze TMB oxidation without H_2_O_2_, as reported in previous studies using a similar synthesis protocol [24]. The volume of MnO_2_ nanoflowers (1 mg/mL) is a key factor influencing sensor performance. To determine the optimal volume, various amounts were tested for labeling target bacteria at 10^5^ CFU/mL, and signal saturation was measured. As shown in Figure 6, increasing the volume from 10 μL to 14 μL improved the saturation value to 0.39, beyond which no significant enhancement was observed. Therefore, 14 μL was selected as the optimal volume for subsequent assays.

#### 3.3.3. Optimization of Mixing Time

**Figure 7:**
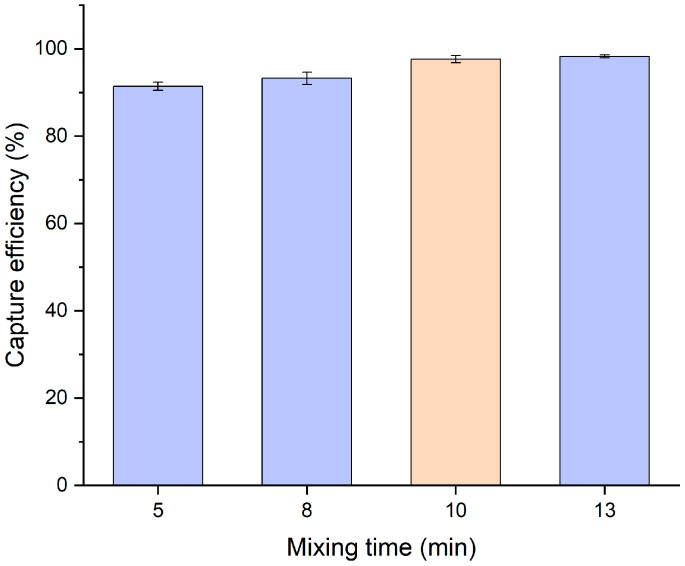
Optimization of mixing time. Error bars represent standard deviations from three independent measurements (N = 3).

Mixing time is critical for the formation of the bead–bacteria–MnO_2_ nanoflower complex. As illustrated in Figure 7, at a target bacterial concentration of 10^5^ CFU/mL, increasing the mixing time from 5 to 13 min enhanced the capture efficiency from 90.5% to 98.5%. However, further extension beyond this point resulted in negligible improvement. Accordingly, 10 min was selected as the optimal mixing time for balancing efficiency and assay duration.

### 3.4. Performance of the Biosensor

**Figure 8:**
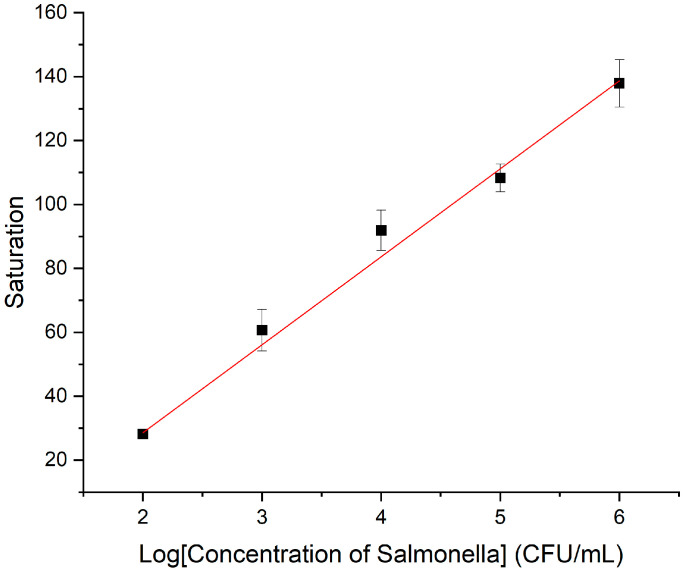
Standard curve construction. Error bars represent standard deviations from three independent measurements (N = 3). Saturation (S) refers to the normalized absorbance of oxidized TMB, calculated as OD_450nm_/OD_max_, where OD_max_ = 1.0 (the plateau value at high bacterial concentration). The plotted range corresponds to 9.5 × 10^1^–9.5 × 10^5^ CFU/mL at the logarithmic scale.

Under optimized conditions, the biosensor was used to detect *Salmonella* over a concentration range of 9.5 × 10^1^ to 9.5 × 10^5^ CFU/mL for standard curve construction. As shown in Figure 8, a strong linear correlation was observed between the saturation signal (S) and the logarithm of bacterial concentration (C), described by the equation:

S = 26.706 log(C) − 21.4233 (R^2^ = 0.9895).

Here, Saturation (S) refers to the normalized absorbance of oxidized TMB, calculated as OD_450nm_/OD_max_, where OD_max_ = 1.0, representing the plateau absorbance value observed at high bacterial concentrations. The limit of detection (LOD) was determined to be 10 CFU/mL, calculated as the concentration corresponding to the saturation signal equal to the mean of the blank group plus three times its standard deviation (n = 10). The limit of detection (LOD) was determined to be 10 CFU/mL.

**Figure 9:**
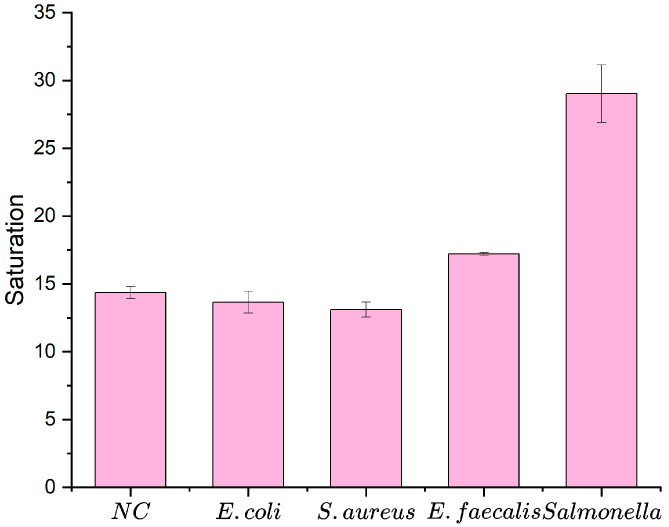
Specificity of the biosensor. Error bars represent standard deviations from three independent measurements (N = 3). Saturation (S) refers to the normalized absorbance of oxidized TMB, calculated as OD_450nm_/OD_max_, where OD_max_ = 1.0 (the plateau value at high bacterial concentration).

Three non-target pathogens—*Listeria monocytogenes* (ATCC 13932), *Escherichia coli* O157:H7 (ATCC 43888), and *Staphylococcus aureus* (CICC 10001) (8.0 × 10^5^ CFU/mL)—were tested alongside *Salmonella* (3.5 × 10^3^ CFU/mL) and mixed samples. As shown in Figure 9, the saturation signals for *Salmonella* and the mixed samples were significantly higher, confirming high specificity.

Spiked pork samples (6.4 × 10^1^ to 2.5 × 10^5^ CFU/mL) were analyzed following China’s national food safety standards (Table 1). The sensor’s results slightly deviated from standard plate counts, likely due to interference from proteins and fats in the matrix. Recovery rates ranged from 94.9% to 125.4%, with a coefficient of variation (CV) of 4.04–6.77%, demonstrating excellent reproducibility.

In addition to assay performance, the long-term stability of key reagents is essential for field deployment. Although specific stability tests were not included in this study, both antibody-functionalized magnetic beads and MnO_2_ nanozymes are known to be compatible with ambient or lyophilized storage. Commercial immunomagnetic beads are routinely supplied in freeze-dried form and maintain high binding activity after prolonged room-temperature storage. Likewise, MnO_2_ nanozymes have demonstrated strong operational stability under repeated use and ambient conditions. For instance, Sun et al. reported that MnO_2_ retained high catalytic activity for multiple cycles of estrogen degradation with minimal activity loss, supporting its robustness in practical applications [25]. These findings suggest that both components can be formulated into lyophilized reagents for cold-chain-free, field-deployable biosensing. In future work, we will further explore reagent stabilization and packaging strategies to enhance environmental durability.

## 4. Conclusions

In this study, we developed a motor-assisted sliding microfluidic colorimetric biosensor for the rapid and sensitive detection of *Salmonella*. The system integrates a sliding microfluidic chip that utilizes expansion and contraction micromixers to enhance reagent mixing and target interaction. By enabling the sequential transfer of dual-antibody sandwich complexes across multiple chambers, the device facilitates targeted bacterial capture, effective washing, and colorimetric signal development. Manganese dioxide nanoflowers were employed as peroxidase-mimicking nanozymes to amplify the colorimetric response.

The biosensor achieved detection within 20 min, with a limit of detection as low as 10 CFU/mL. In spiked pork samples, it demonstrated recovery rates between 94.9% and 125.4%, with coefficients of variation below 6.8%, indicating high analytical accuracy and reproducibility. Owing to its rapid response, low cost, simple operation, and excellent sensitivity, this biosensor shows strong potential for the on-site detection of foodborne pathogens in both routine monitoring and emergency response applications.

## Figures and Tables

**Table 1:** The recovery of *Salmonella* in spiked pork samples (N = 3).

Spike Concentration (CFU/mL)	Detected Concentration (CFU/mL)	Recovery (%)	RSD (%)
64	63.8	99.7	5.76
640	607.4	94.9	5.60
2600	3261.6	125.4	6.77
260,000	28,252.0	108.7	4.04

## Data Availability

The original contributions presented in this study are included in the article. Further inquiries can be directed to the corresponding author(s).
